# Supplementary material for: A Likelihood Approach for Real-Time Calibration of Stochastic Compartmental Epidemic Models
Source: PLoS Comput Biol. 2017 Jan 17;13(1):e1005257. doi: 10.1371/journal.pcbi.1005257 (PMC5240920; doi:10.1371/journal.pcbi.1005257)
Supplement: S1 File — (TAR.GZ) [file pcbi.1005257.s014.tar.gz › HSPH_Online-SI-Revision/Additions/IPoi10/n100-extreme_noisy/n100-extreme_noisy_BENCH2_table8.pdf]

1	newly 3 weeks spec	526.162	1098.07	{530., 1890.}	110.612	0.276141	newly 3 weeks cumul	2501.43	4697.13	{2191., 7919.}	92.1677	0.244785	newly 5 weeks spec	280.291	563.471	{265., 968.}	103.944	0.26413	newly 5 weeks cumul	3116.21	6053.26	{2801., 10 255.}	97.8206	0.254633	newly 46 weeks cumul	6824.91	7505.28	{3525., 12 864.}	40.5255	0.160031
2	newly 3 weeks spec	761.	587.206	{254., 1131.}	52.1473	0.272837	newly 3 weeks cumul	3077.	2373.41	{1094., 4704.}	51.2713	0.268127	newly 5 weeks spec	441.	340.222	{152., 635.}	52.1362	0.273561	newly 5 weeks cumul	4068.	3159.37	{1405., 6276.}	51.3836	0.267536	newly 42 weeks cumul	5247.	4228.37	{1894., 8361.}	51.438	0.259521
3	newly 3 weeks spec	277.	448.676	{196., 591.}	75.8654	0.190048	newly 3 weeks cumul	1321.	2023.65	{909., 2771.}	68.3373	0.178218	newly 5 weeks spec	122.	213.956	{90., 283.}	85.4749	0.204449	newly 5 weeks cumul	1607.	2548.01	{1140., 3462.}	72.423	0.184597	newly 47 weeks cumul	1853.	3026.72	{1341., 4081.}	75.9222	0.189259
4	newly 3 weeks spec	75.	264.762	{107., 496.}	253.27	0.462859	newly 3 weeks cumul	495.	1470.43	{615., 2641.}	198.307	0.393847	newly 5 weeks spec	26.	94.9841	{39., 163.}	266.056	0.474731	newly 5 weeks cumul	564.	1727.59	{712., 3081.}	207.441	0.406234	newly 51 weeks cumul	589.	1869.83	{769., 3308.}	218.412	0.420816
5	newly 3 weeks spec	383.	706.392	{294., 927.}	95.8472	0.214157	newly 3 weeks cumul	1884.	3111.14	{1338., 4164.}	81.6133	0.202201	newly 5 weeks spec	164.	342.176	{143., 432.}	116.027	0.236635	newly 5 weeks cumul	2324.	3949.26	{1676., 5217.}	85.4102	0.20546	newly 48 weeks cumul	2679.	4723.85	{2002., 6238.}	90.3872	0.210203
6	newly 3 weeks spec	620.	591.761	{279., 1036.}	45.7179	0.20159	newly 3 weeks cumul	2591.	2533.56	{1172., 4522.}	45.2879	0.194906	newly 5 weeks spec	303.	304.085	{140., 545.}	47.204	0.198567	newly 5 weeks cumul	3314.	3259.59	{1528., 5810.}	45.5103	0.194629	newly 46 weeks cumul	4005.	4025.66	{1874., 7169.}	46.0625	0.192493
7	newly 3 weeks spec	425.	1047.	{547., 1827.}	146.353	0.349378	newly 3 weeks cumul	2093.	4316.6	{2268., 7504.}	106.701	0.275652	newly 5 weeks spec	238.	574.672	{293., 980.}	141.459	0.340018	newly 5 weeks cumul	2658.	5663.97	{2940., 9795.}	113.462	0.288932	newly 44 weeks cumul	3121.	7326.72	{3848., 12 611.}	134.756	0.328621
8	newly 3 weeks spec	611.	929.194	{352., 1607.}	75.1765	0.212281	newly 3 weeks cumul	2634.	3954.49	{1488., 6899.}	72.7382	0.208371	newly 5 weeks spec	315.	489.836	{177., 836.}	79.403	0.219775	newly 5 weeks cumul	3371.	5112.45	{1944., 8882.}	74.204	0.210294	newly 46 weeks cumul	4133.	6346.7	{2381., 10 937.}	75.646	0.211236
9	newly 3 weeks spec	509.	1254.04	{547., 1933.}	147.12	0.315383	newly 3 weeks cumul	2442.	5277.06	{2322., 8155.}	118.457	0.272731	newly 5 weeks spec	256.	664.243	{283., 1037.}	160.352	0.332494	newly 5 weeks cumul	3053.	6851.76	{2963., 10 627.}	126.288	0.284191	newly 46 weeks cumul	3616.	8516.23	{3690., 13 187.}	136.742	0.299402
10	newly 3 weeks spec	32.	160.018	{64., 228.}	400.055	0.607911	newly 3 weeks cumul	165.	873.789	{370., 1257.}	429.569	0.634383	newly 5 weeks spec	7.	55.3684	{17., 91.}	690.977	0.794049	newly 5 weeks cumul	184.	1023.63	{437., 1468.}	456.322	0.6554	newly 50 weeks cumul	192.	1107.49	{481., 1586.}	476.818	0.671002
11	newly 3 weeks spec	582.	837.779	{396., 1559.}	63.0812	0.173641	newly 3 weeks cumul	2565.	3420.46	{1641., 6460.}	57.3598	0.172018	newly 5 weeks spec	318.	461.456	{210., 910.}	65.7279	0.181914	newly 5 weeks cumul	3307.	4501.35	{2138., 8515.}	59.0895	0.173702	newly 44 weeks cumul	4044.	5829.31	{2766., 10 971.}	63.2553	0.17516
12	newly 3 weeks spec	488.	1371.19	{490., 2029.}	182.813	0.320573	newly 3 weeks cumul	2113.	5531.71	{2109., 8585.}	163.629	0.309806	newly 5 weeks spec	223.	754.929	{260., 1112.}	238.994	0.37812	newly 5 weeks cumul	2630.	7312.37	{2757., 11 222.}	179.224	0.325263	newly 45 weeks cumul	3083.	9374.51	{3402., 14 245.}	204.65	0.353386
13	newly 3 weeks spec	1177.	878.015	{526., 1341.}	40.0575	0.21526	newly 3 weeks cumul	4644.	3520.	{2136., 5362.}	38.9233	0.206851	newly 5 weeks spec	679.	502.354	{280., 793.}	40.929	0.221327	newly 5 weeks cumul	6187.	4688.08	{2823., 7182.}	39.2361	0.208607	newly 43 weeks cumul	8302.	6276.2	{3683., 9625.}	39.6485	0.211136
14	newly 3 weeks spec	9.	545.938	{272., 961.}	5965.98	1.73607	newly 3 weeks cumul	64.	2577.4	{1409., 4452.}	3927.19	1.56031	newly 5 weeks spec	5.	243.338	{128., 409.}	4766.77	1.64187	newly 5 weeks cumul	74.	3191.09	{1723., 5518.}	4212.29	1.58995	newly 48 weeks cumul	86.	3677.97	{2010., 6377.}	4176.71	1.5861
15	newly 3 weeks spec	73.	481.262	{253., 684.}	559.262	0.760759	newly 3 weeks cumul	382.	2254.17	{1211., 3243.}	490.097	0.71486	newly 5 weeks spec	19.	217.185	{116., 302.}	1043.08	0.996559	newly 5 weeks cumul	449.	2794.97	{1509., 4071.}	522.488	0.737112	newly 48 weeks cumul	476.	3241.25	{1724., 4783.}	580.934	0.775217
16	newly 3 weeks spec	111.	350.94	{167., 534.}	216.162	0.440186	newly 3 weeks cumul	653.	1643.52	{840., 2469.}	152.419	0.3425	newly 5 weeks spec	31.	154.358	{70., 230.}	397.93	0.628182	newly 5 weeks cumul	748.	2030.06	{1038., 3040.}	171.722	0.37259	newly 48 weeks cumul	812.	2337.97	{1177., 3444.}	188.119	0.397266
17	newly 3 weeks spec	848.	1335.32	{699., 2565.}	65.9716	0.181753	newly 3 weeks cumul	3379.	5290.35	{2824., 9885.}	64.5327	0.179835	newly 5 weeks spec	459.	762.091	{394., 1503.}	73.0178	0.192688	newly 5 weeks cumul	4480.	7068.5	{3712., 13 379.}	65.7261	0.181567	newly 43 weeks cumul	5685.	9430.18	{4919., 17 842.}	72.2683	0.193431
18	newly 3 weeks spec	371.	1263.52	{693., 2111.}	240.573	0.491985	newly 3 weeks cumul	1789.	5160.9	{2900., 8681.}	188.48	0.421349	newly 5 weeks spec	168.	712.607	{389., 1179.}	324.171	0.585759	newly 5 weeks cumul	2207.	6823.92	{3844., 11 469.}	209.194	0.450848	newly 44 weeks cumul	2601.	8912.98	{5016., 14 986.}	242.675	0.494359
19	newly 3 weeks spec	522.	1098.73	{516., 1695.}	113.135	0.275973	newly 3 weeks cumul	2300.	4681.05	{2203., 6924.}	106.127	0.265019	newly 5 weeks spec	225.	571.217	{278., 852.}	154.793	0.346854	newly 5 weeks cumul	2858.	6043.63	{2856., 8960.}	113.54	0.277984	newly 46 weeks cumul	3344.	7498.3	{3556., 10 946.}	125.6	0.299041
20	newly 3 weeks spec	31.	213.836	{97., 403.}	589.794	0.770769	newly 3 weeks cumul	168.	1173.3	{553., 2127.}	598.39	0.777087	newly 5 weeks spec	4.	73.9672	{34., 140.}	1749.18	1.19873	newly 5 weeks cumul	185.	1376.98	{644., 2496.}	644.315	0.805539	newly 51 weeks cumul	187.	1488.11	{713., 2653.}	695.783	0.834472
21	newly 3 weeks spec	203.	669.935	{275., 751.}	230.145	0.383494	newly 3 weeks cumul	1027.	2994.52	{1326., 3655.}	192.012	0.350787	newly 5 weeks spec	76.	310.839	{120., 338.}	309.295	0.45729	newly 5 weeks cumul	1233.	3761.19	{1669., 4490.}	205.455	0.363741	newly 48 weeks cumul	1341.	4393.48	{1944., 5125.}	227.921	0.3892
22	newly 3 weeks spec	138.	607.609	{244., 1050.}	340.297	0.520461	newly 3 weeks cumul	788.	2854.42	{1189., 4884.}	262.236	0.444646	newly 5 weeks spec	76.	265.672	{113., 437.}	249.568	0.416111	newly 5 weeks cumul	953.	3522.69	{1452., 5968.}	269.642	0.451719	newly 49 weeks cumul	1046.	4038.06	{1666., 6792.}	286.048	0.469131
23	newly 3 weeks spec	646.	1103.87	{483., 1957.}	81.0458	0.214229	newly 3 weeks cumul	2717.	4361.84	{1959., 7778.}	72.278	0.19907	newly 5 weeks spec	343.	644.857	{289., 1158.}	94.1136	0.232512	newly 5 weeks cumul	3563.	5847.43	{2618., 10 474.}	74.9257	0.202493	newly 42 weeks cumul	4442.	7872.3	{3479., 14 124.}	85.2415	0.218819
24	newly 3 weeks spec	888.	1112.	{485., 1602.}	54.0943	0.170762	newly 3 weeks cumul	3507.	4364.39	{2085., 6320.}	51.4512	0.16808	newly 5 weeks spec	441.	676.554	{305., 948.}	70.8576	0.18228	newly 5 weeks cumul	4589.	5912.36	{2778., 8517.}	54.6664	0.171013	newly 42 weeks cumul	5895.	8044.32	{3717., 11 367.}	60.1284	0.175319
25	newly 3 weeks spec	135.	1022.42	{475., 1589.}	657.35	0.813643	newly 3 weeks cumul	801.	4302.17	{2005., 6437.}	437.1	0.668288	newly 5 weeks spec	46.	534.563	{240., 790.}	1062.09	0.997336	newly 5 weeks cumul	936.	5585.16	{2583., 8340.}	496.705	0.712608	newly 45 weeks cumul	1018.	7000.42	{3231., 10 438.}	587.664	0.773064
26	newly 3 weeks spec	1048.	1181.39	{516., 2024.}	50.4087	0.187389	newly 3 weeks cumul	4173.	4688.01	{2107., 8163.}	48.5466	0.181377	newly 5 weeks spec	644.	683.746	{304., 1136.}	50.4009	0.196121	newly 5 weeks cumul	5651.	6276.04	{2772., 10 847.}	48.787	0.183567	newly 43 weeks cumul	7533.	8406.96	{3699., 14 493.}	49.7067	0.185119
27	newly 3 weeks spec	806.	609.603	{312., 1176.}	46.1703	0.250893	newly 3 weeks cumul	3332.	2552.	{1337., 4871.}	45.7789	0.245347	newly 5 weeks spec	451.	340.397	{182., 665.}	46.6512	0.252918	newly 5 weeks cumul	4401.	3354.29	{1741., 6354.}	45.9434	0.247017	newly 44 weeks cumul	5811.	4316.1	{2221., 8268.}	46.271	0.253562
28	newly 3 weeks spec	568.	399.356	{173., 832.}	54.1418	0.308335	newly 3 weeks cumul	2482.	1867.25	{849., 3792.}	52.4249	0.283103	newly 5 weeks spec	266.	176.932	{75., 368.}	55.5945	0.329542	newly 5 weeks cumul	3132.	2312.29	{1035., 4687.}	52.9509	0.28995	newly 48 weeks cumul	3853.	2665.	{1183., 5410.}	54.1361	0.310259
29	newly 3 weeks spec	769.	1299.86	{730., 2158.}	73.0259	0.20526	newly 3 weeks cumul	3241.	5161.54	{2931., 8581.}	64.7222	0.18883	newly 5 weeks spec	412.	762.523	{408., 1243.}	87.7894	0.234637	newly 5 weeks cumul	4213.	6916.03	{3894., 11 419.}	68.954	0.197293	newly 42 weeks cumul	5339.	9292.15	{5184., 15 415.}	77.2853	0.21366
30	newly 3 weeks spec	278.	238.056	{100., 391.}	48.2217	0.234247	newly 3 weeks cumul	1314.	1246.72	{513., 1984.}	47.8284	0.210674	newly 5 weeks spec	87.	93.2254	{39., 155.}	51.6108	0.199202	newly 5 weeks cumul	1559.	1487.2	{608., 2347.}	47.9542	0.209677	newly 51 weeks cumul	1722.	1632.21	{672., 2602.}	47.9732	0.211144
31	newly 3 weeks spec	58.	186.766	{82., 377.}	222.872	0.427703	newly 3 weeks cumul	360.	1078.42	{443., 2119.}	199.805	0.399786	newly 5 weeks spec	22.	62.3906	{28., 134.}	185.866	0.382547	newly 5 weeks cumul	413.	1248.77	{517., 2483.}	202.622	0.403754	newly 52 weeks cumul	442.	1331.66	{557., 2642.}	201.598	0.401833
32	newly 3 weeks spec	101.	444.412	{245., 631.}	340.012	0.589192	newly 3 weeks cumul	549.	2126.09	{1164., 3140.}	287.266	0.535397	newly 5 weeks spec	30.	192.426	{107., 275.}	541.422	0.753051	newly 5 weeks cumul	633.	2615.84	{1433., 3846.}	313.245	0.563347	newly 49 weeks cumul	682.	2991.66	{1651., 4391.}	338.66	0.589249
33	newly 3 weeks spec	918.	625.288	{294., 1150.}	43.9755	0.260763	newly 3 weeks cumul	3832.	2758.31	{1381., 5072.}	41.9076	0.239586	newly 5 weeks spec	479.	306.983	{143., 565.}	45.5575	0.281219	newly 5 weeks cumul	4959.	3500.69	{1724., 6498.}	42.4498	0.245781	newly 47 weeks cumul	6261.	4212.19	{2065., 7816.}	44.0061	0.262072
34	newly 3 weeks spec	843.	955.	{524., 1823.}	42.237	0.164063	newly 3 weeks cumul	3518.	3831.43	{2141., 7229.}	39.6005	0.159722	newly 5 weeks spec	471.	552.058	{280., 1083.}	44.6198	0.167751	newly 5 weeks cumul	4638.	5111.	{2803., 9772.}	40.265	0.160528	newly 42 weeks cumul	6028.	6846.81	{3747., 13 174.}	41.6164	0.160852
35	newly 3 weeks spec	693.	662.712	{322., 1110.}	44.8926	0.192956	newly 3 weeks cumul	3121.	2925.91	{1418., 4817.}	43.8981	0.193394	newly 5 weeks spec	338.	323.5	{153., 555.}	44.5446	0.193075	newly 5 weeks cumul	3977.	3715.03	{1799., 6125.}	44.3169	0.19585	newly 47 weeks cumul	4760.	4474.42	{2166., 7386.}	44.6091	0.195652
36	newly 3 weeks spec	635.	343.717	{158., 569.}	53.9554	0.356438	newly 3 weeks cumul	2904.	1466.67	{685., 2421.}	54.9346	0.374641	newly 5 weeks spec	364.	177.6	{78., 311.}	56.456	0.397144	newly 5 weeks cumul	3816.	1890.22	{869., 3149.}	55.4529	0.382107	newly 45 weeks cumul	4844.	2347.17	{1070., 3957.}	56.0687	0.390653
37	newly 3 weeks spec	121.	471.127	{216., 917.}	289.361	0.524694	newly 3 weeks cumul	634.	2318.49	{1049., 4447.}	265.693	0.497582	newly 5 weeks spec	49.	193.762	{88., 353.}	295.432	0.52808	newly 5 weeks cumul	759.	2810.22	{1265., 5434.}	270.253	0.502607	newly 50 weeks cumul	824.	3149.94	{1402., 6077.}	282.274	0.515961
38	newly 3 weeks spec	12.	508.585	{220., 930.}	4138.21	1.55214	newly 3 weeks cumul	113.	2539.	{1122., 4731.}	2146.9	1.27905	newly 5 weeks spec	3.	212.523	{90., 389.}	6984.1	1.77348	newly 5 weeks cumul	121.	3082.52	{1354., 5768.}	2447.54	1.33296	newly 50 weeks cumul	126.	3458.69	{1537., 6481.}	2644.99	1.3653
39	newly 3 weeks spec	302.	1303.08	{664., 1913.}	331.483	0.579966	newly 3 weeks cumul	1471.	5311.46	{2805., 7800.}	261.078	0.506714	newly 5 weeks spec	115.	738.476	{379., 1105.}	542.153	0.750387	newly 5 weeks cumul	1796.	7034.43	{3723., 10 306.}	291.672	0.540968	newly 44 weeks cumul	2002.	9229.13	{4816., 13 585.}	360.995	0.610127
40	newly 3 weeks spec	529.	602.783	{298., 1011.}	41.8746	0.155946	newly 3 weeks cumul	2325.	2588.7	{1268., 4439.}	40.4789	0.154598	newly 5 weeks spec	275.	318.9	{156., 507.}	42.3273	0.15563	newly 5 weeks cumul	2963.	3346.67	{1610., 5741.}	41.0395	0.154648	newly 45 weeks cumul	3697.	4154.48	{2010., 7009.}	41.1744	0.155986
41	newly 3 weeks spec	253.	618.029	{327., 1136.}	145.001	0.338057	newly 3 weeks cumul	1228.	2731.04	{1459., 4898.}	123.737	0.301889	newly 5 weeks spec	114.	303.456	{153., 585.}	166.576	0.373346	newly 5 weeks cumul	1511.	3467.54	{1852., 6298.}	130.54	0.313718	newly 46 weeks cumul	1685.	4163.	{2256., 7654.}	147.783	0.343596
42	newly 3 weeks spec	23.	139.672	{59., 218.}	507.268	0.677843	newly 3 weeks cumul	166.	758.746	{309., 1204.}	357.076	0.556001	newly 5 weeks spec	8.	51.5373	{22., 77.}	544.216	0.699936	newly 5 weeks cumul	186.	895.925	{367., 1411.}	381.68	0.578869	newly 50 weeks cumul	187.	974.075	{400., 1557.}	420.896	0.612357
43	newly 3 weeks spec	681.	1652.89	{821., 3346.}	143.88	0.33556	newly 3 weeks cumul	3118.	6534.62	{3433., 12 926.}	111.311	0.278345	newly 5 weeks spec	377.	969.54	{480., 2002.}	157.972	0.353334	newly 5 weeks cumul	4043.	8772.37	{4548., 17 453.}	118.49	0.290803	newly 43 weeks cumul	5018.	11 852.4	{6056., 24 002.}	137.264	0.323155
44	newly 3 weeks spec	174.	179.545	{92., 247.}	40.9962	0.160831	newly 3 weeks cumul	824.	892.561	{472., 1200.}	40.5358	0.149933	newly 5 weeks spec	61.	72.2727	{37., 100.}	45.0075	0.152026	newly 5 weeks cumul	988.	1082.24	{582., 1498.}	41.0134	0.149568	newly 49 weeks cumul	1084.	1213.24	{651., 1662.}	41.6247	0.148125
45	newly 3 weeks spec	45.	406.052	{243., 609.}	802.337	0.920918	newly 3 weeks cumul	244.	1915.52	{1129., 2750.}	685.048	0.860292	newly 5 weeks spec	9.	179.414	{105., 273.}	1893.49	1.25827	newly 5 weeks cumul	276.	2367.47	{1393., 3404.}	757.777	0.898184	newly 48 weeks cumul	280.	2733.4	{1612., 3882.}	876.213	0.953975
46	newly 3 weeks spec	223.	1000.61	{375., 1440.}	348.706	0.532407	newly 3 weeks cumul	1040.	4274.34	{1824., 6449.}	310.995	0.522151	newly 5 weeks spec	61.	514.457	{190., 685.}	743.372	0.783337	newly 5 weeks cumul	1223.	5512.9	{2295., 8132.}	350.769	0.552767	newly 48 weeks cumul	1322.	6684.33	{2730., 9569.}	405.622	0.594388
47	newly 3 weeks spec	132.	593.478	{244., 1217.}	349.605	0.546696	newly 3 weeks cumul	614.	2913.14	{1222., 5953.}	374.454	0.575046	newly 5 weeks spec	43.	244.188	{100., 502.}	467.88	0.645379	newly 5 weeks cumul	740.	3538.57	{1456., 7242.}	378.184	0.577209	newly 50 weeks cumul	804.	3976.87	{1629., 8179.}	394.636	0.590777
48	newly 3 weeks spec	186.	554.286	{222., 1008.}	198.942	0.377826	newly 3 weeks cumul	969.	2748.84	{1126., 4985.}	184.844	0.36099	newly 5 weeks spec	49.	230.73	{85., 425.}	370.878	0.567935	newly 5 weeks cumul	1115.	3339.9	{1358., 6071.}	200.317	0.381548	newly 50 weeks cumul	1182.	3748.02	{1527., 6807.}	217.591	0.40462
49	newly 3 weeks spec	8.	82.4688	{41., 138.}	930.859	0.966591	newly 3 weeks cumul	68.	498.313	{259., 787.}	632.813	0.821266	newly 5 weeks spec	3.	25.6563	{12., 44.}	755.208	0.87786	newly 5 weeks cumul	72.	569.156	{291., 908.}	690.495	0.853804	newly 52 weeks cumul	76.	596.063	{304., 939.}	684.293	0.850139
50	newly 3 weeks spec	733.	874.083	{507., 1422.}	39.8287	0.14614	newly 3 weeks cumul	2944.	3610.89	{2062., 5831.}	40.9015	0.145861	newly 5 weeks spec	408.	494.139	{279., 791.}	41.5101	0.149974	newly 5 weeks cumul	3889.	4768.53	{2704., 7668.}	41.1195	0.146643	newly 43 weeks cumul	4985.	6211.56	{3513., 9960.}	42.3231	0.14866
